# Introduction of a modified single stage reconstruction technique of male penopubic epispadias

**DOI:** 10.1186/s12894-022-01089-2

**Published:** 2022-08-29

**Authors:** Masoud Bitaraf, Pouya Mahdavi Sharif, Parham Torabinavid, Abdol-Mohammad Kajbafzadeh

**Affiliations:** Pediatric Urology and Regenerative Medicine Research Center, Gene, Cell and Tissue Research Institute, Children’s Medical Center, Tehran University of Medical Science, No. 62, Dr. Qarib’s St, Keshavarz Blvd, Tehran, 14194 33151 Iran

**Keywords:** Epispadias, Urinary incontinence, Urogenital abnormalities, Bladder exstrophy and epispadias complex, Reconstructive surgical procedures, Male urogenital diseases, Erectile dysfunction, Sexual health, Surgical flaps

## Abstract

**Objective:**

To represent the long-term outcomes of our modified single-stage technique for the reconstruction of isolated penopubic epispadias in male patients.

**Patients and methods:**

Data from 113 patients were obtained from bladder-exstrophy-epispadias database of our tertiary center. A total of seven boys with isolated penopubic epispadias with no prior history of surgery and any other anomaly underwent our modified surgical approach from February 1997 to September 2019. The mean ± SD age at surgery was 6.5 ± 2.4 years. Volitional voiding status and cosmetic appearance were evaluated at each follow-up interval. Postoperative follow-up was performed at quarterly intervals in the first year and once a year in subsequent years.

**Results:**

The mean ± SD of follow-up was 8.5 ± 6 years. All boys who were incontinent achieved urinary control and the ability of normal transurethral micturition following the surgery. Four boys became completely dry, and the other three attained social dryness. Postoperative mean (SD) bladder capacity was significantly increased from 54.5 (11) to 124 (40.0) within 6 months, and to 194 (47.5) at 18 months after surgery. Dorsal curvature has been resolved in all cases, and no postoperative complications were noted except for surgical site infection in one patient treated with antibiotics and bilateral vesicourethral reflux resolved after injection of bulking agents. Four patients had normal erectile function and ejaculation, while the others have not reached puberty yet. Moreover, none of the patients developed urethrocutaneous fistula, stricture, or penile ischemia.

**Conclusion:**

The present findings suggest the safety and effectiveness of the combination of single-stage urethro-genitoplasty, bladder neck plication, and fat pad pedicled flap in management of boys with isolated penopubic epispadias that can lead to the achievement of urinary control, acceptable sexual function, and cosmetically satisfactory genitalia. Minimal morbidity, low complication rate, and promising outcomes are essential factors, supporting the notion of introducing this technique as a valid option for management of this entity.

## Introduction

Male epispadias, a rare congenital dislocation of the urethral meatus on the dorsum of the penis, is of the mildest form of a broad spectrum of urogenital anomalies known as bladder-exstrophy-epispadias complex (BEEC) [1]. Although the etiology of this entity is not completely understood, disruption of mesenchymal migration due to the early invasion of the cloacal membrane around five weeks of gestation may proceed to the development of such an anomaly [2]. The scarcity of epispadias is reported as 1 in 117,000 male live births [3]. However, given that the glandular epispadias with obscured meatus beneath the prepuce is not well diagnosed, the prevalence seems to be higher [4]. Penopubic epispadias (PPE) is the most common as well as the most severe form of epispadias. Lack of urethral tubularization and failure in urethral plate closure leads to development of a broad mucosal strip on the penile dorsum and exposure of a patulous bladder neck (BN) and posterior urethra, along with sphincter incompetence in penopubic type. This variant is also associated with variable degrees of urinary incontinence (up to 70%), penile dorsal curvature, and an unpleasant appearance [1, 4, 5].

The concerns regarding the management of isolated PPE have recently shifted to improving the health-related quality of life. Lack of urinary continence, dissatisfaction with genitalia, and impaired sexual function are essential factors in such patients, increasing the risk of psychosocial harm, sexual health disturbance, impairment of body perception, and mental disorders. Therefore, achieving volitional voiding ability, reconstructing a cosmetically acceptable penis, and maintaining sexual function are paramount [6–8]. Although surgical approaches have made considerable progress with promising outcomes in recent years, restoring continence has remained a major concern in these patients. Moreover, multi-stage surgeries and application of bladder neck reconstruction (BNR) are accompanied with conflicting results and high morbidity [8–11].

Herein, we represent the long-term outcomes of our single-surgeon single-center experience with modified surgical technique as a novel approach for the management of boys with isolated PPE. A technique in which we do not open the bladder neither for bladder neck plication nor for urethral reimplantation.

### Patients and methods

This study is approved by the Committee of Human Research and the Institutional Review Board at our university and all methods were carried out in accordance with the declaration of Helsinky. After explaining all the potential risks and benefits of this technique and the possible post-surgical harms, informed consent was obtained from the legal guardians or parents of the included children. Our BEEC database was reviewed for the medical records of boys with PPE. Patients who underwent our current modified surgical approach were included, and those who had other anomalies in the genitourinary system, or those with previous unsuccessful surgery, were excluded from this study. All patients underwent a thorough physical examination along with the assessment of incontinence severity. Subsequently, voiding cystourethrography (VCUG); for presence of reflux and to estimate the bladder capacity, ultrasonography, urine analysis, urine culture, and 3D-CT scan of pelvic bone were performed for each child in order to depict 3 dimensional pelvic bone anatomy and measure all bony angles and bone dimensions and bone distances of pelvis. Post-operative continence status was categorized based on a previously defined classification. Briefly, totally continent patients (Grade 0) were considered as those who were totally dry at night with dry intervals of ≥ 6 h during the day, and considered as occasionally wet (Grade 1) if they used pads for at least once a week or had infrequent episodes of incontinence. Lastly, those with dry intervals of < 3 h and those with no dry period was considered as frequently wet (Grade 2), and totally incontinent (Grade 3), respectively. Moreover, children who had dry intervals of ≥ 3 h during the day were considered socially dry patients (grade 0 and 1).

### Surgical technique

**Fig. 1:**
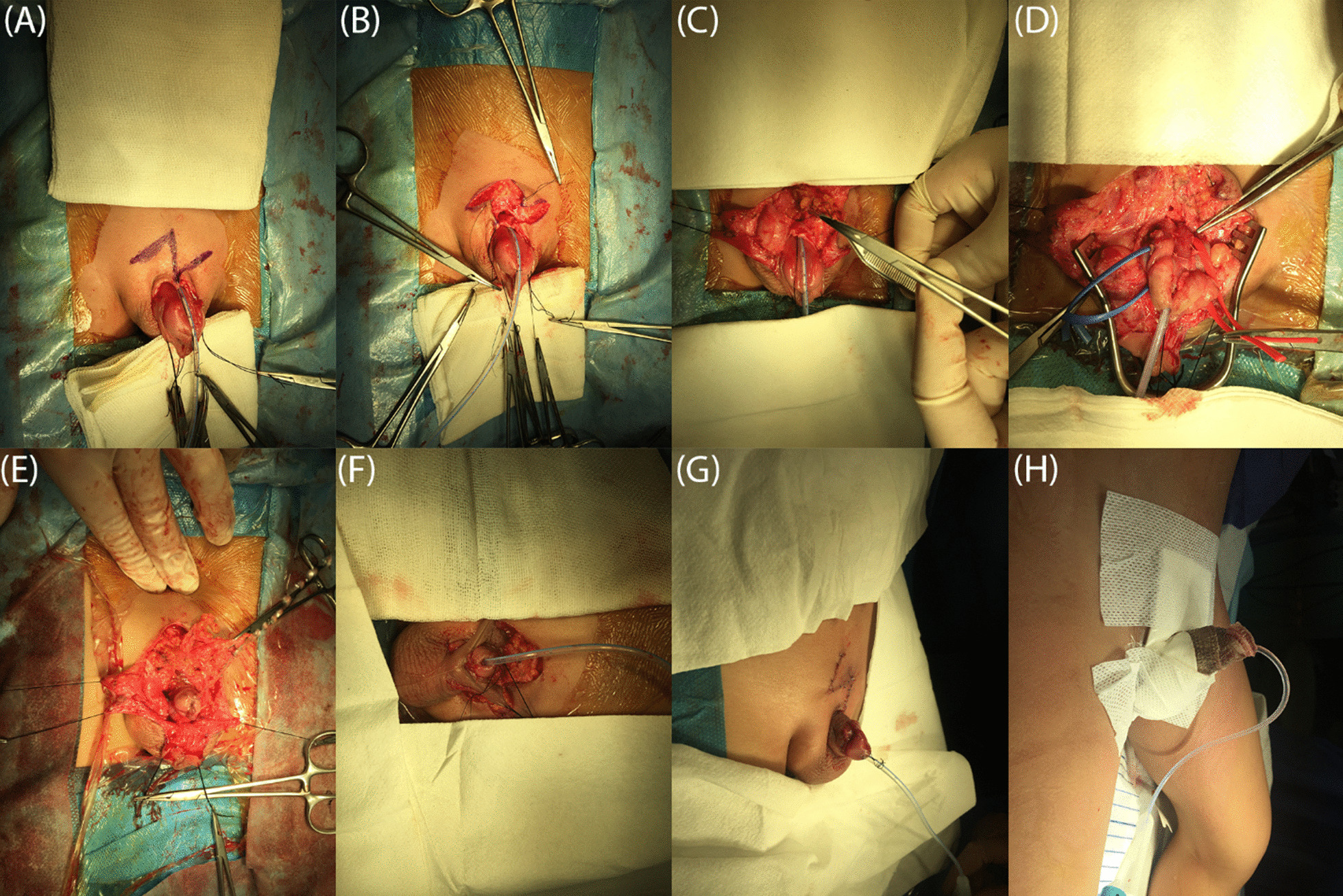
Surgical technique; **a**, **b** Z-like incision at suprapubic to expose bladder neck and verumontanum, **c** bladder neck plication, **d** isolation of corporal bodies using vascular tapes, **e** manual compression of bladder to evaluate bladder neck competency, **f** recatheterization using a smaller catheter, **g** skin closure and catheter fixation, **h** after dressing

Following the general anesthesia, cystoscopy was performed in all children, evaluating the BN configuration, sphincter competency, and ureteral orifices. After that, using polypropylene 4/0, two traction sutures were placed on both sides of the glans to circumscribe the urethral plate and underlying corpus spongiosum (urethral wedge) margins. The reverse MAGPI (meatal advancement and glanuloplasty, IPGAM) incision was done at the distal glandular end of the urethral wedge. After defining the urethral wedge boundaries with a surgical marker, an inverted U-shaped incision was made along the lateral margins of the urethral wedge, starting from the distal portion, continued up to the BN. Circumcision incision was performed on the penile ventral aspect, confluence the U-shaped incision of the urethra on the dorsum. By avoiding complete penile degloving, the skin was dissected off the penis with mesothelial sparing at the median and paramedian area where the corpus spongiosum is located, and the buck's fascia has just stopped at its edges. The main reason was to preserve a greater blood supply to the corpus spongiosum and the adjacent structures, as it is the main source of blood supply to the urethra, precipitating the healing process and preventing further tissue hypoxia. The urethral wedge was proximally dissected off the corpora cavernosa up to the BN and further to the pubic bone rami. It bears mentioning that complete preservation of the corpus spongiosum should be performed carefully. Laterally dislocated neurovascular bundles (NVBs) were meticulously dissected off the corporal bodies. The corporal bodies were separated distally up to the glans, except for the distal 1 cm that was kept attached. A total of five vascular tapes were applied to isolate the structures at each separation step, two for corporal bodies, two for NVBs, and one for the urethral wedge. Skin and the underlying tissues were incised in a Z-like fashion at the suprapubic area to expose the BN and verumontanum. BN was plicated, avoiding any neck or bladder mucosa injury, following Kelly’s plication technique, using non-absorbable sutures, which led to the inversion of the mucosa back to the bladder. According to a previously published article, non-absorbable sutures are superior to absorbable ones, with seemingly better outcomes. A 10 Fr urethral catheter was placed at the BN and the posterior urethra. Tubularization started from BN down to the verumontanum. The bladder was then fulfilled with normal saline to evaluate the BN competency by manually compressing the bladder after removing the catheter. Bladder neck plication (BNP) was considered successful when the urine flowed following compression and stopped after decompression. The key point was avoiding mucosal disturbance and assessment of BN resistance following the plication. The catheter was replaced, two layers, including the urethral plate and underlying corpus spongiosum were tubularized around the 10 Fr catheter up to the distal 2 cm of the glans using 6-0 running sutures. Tubularization was continued up to the meatus. Excessive atypical skin of the glans wings was trimmed off. A normal conical glandular appearance and a ventrally located meatus were achieved following a two-layered closure of the glans wings using 7-0 PDS^®^ (polydioxanone) sutures during the glanuloplasty procedure. Repair of the chordee was performed with interrupted suturing of the reapproximated medially rotated corporal bodies at the dorsal apex, leading to placement of the neo-urethra on the ventral aspect, chordee correction, and placing the NVBs to their original anatomic location. The appearance of the glans was assessed from now on; if it got pale, the blood supply sufficiency was examined by using a diabetic lancet device on the glans. In case of an insufficient blood supply, sutures of the corporal bodies were replaced with looser stitches. Two wide-based long fat pad pedicled flaps were taken from the subcutaneous fats that lies on the rectus muscles sheath. Each side's pedicled fat pad slings passed over the contralateral corporal body cross wisely and connected to the corpus spongiosum on the ventral aspect integrating the three main structures of the penis in a ring fashion at the penile base level with the aim of mimicking the function of an external sphincter. Besides, it is worth noting that the external sphincter plate, located at the distal end of the verumontanum, was intensely protected from invasion, traumatization, and devascularization during each step. So far, the BN is plicated, the corporal bodies are sutured together, and both are fixed in a pedicled fat pad ring along with the ventrally located neo-urethra. The 10 Fr urethral catheter was then removed; following a successful manual examination of the urine flow, neo-urethra was re-catheterized using 8 Fr. urethral catheter, fixed to the glans with two sutures. Recatheterization was performed to examine the competency of the neo-urethra as well as reducing the risk of urethral ischemia regarding the existing peritubular tissue edema. Therefore, three main factors were evaluated during the surgery, the competence of the neo-urethra that was evaluated with recatheterization, BN competency that was evaluated following manual compression after decatheterization, and the ischemic state and blood sufficiency of the penis that was evaluated by using a diabetic lancet device. Finally, the skin was reconfigured using Z-plasty (Fig. 1).

### Post-op and follow-up

Patients were discharged five days after surgery with antibiotic prophylaxis, and the urinary catheter was removed on the 14th post-operative day. Thereafter, a comprehensive evaluation at each visit, including continence status, development of possible complications, clinical examination, and genitalia size, were performed at intervals of quarterly in the first year and once a year in subsequent years. Moreover, uroflowmetry was performed at 1, 6, and 18 months after the surgery. Besides, genitourinary ultrasonography, VCUG, and urine analysis were performed at intervals of 6 months for the first year and annually thereafter. Patients' sexual health, including erectile function, quality of ejaculation, penile size, and dorsal curvature, were also assessed as soon as they reached puberty.

For data analysis, data with *P *< 0.05 was considered statistically significant. The paired t-test was used to evaluate the changes that occurred in bladder capacity during the follow-up intervals.

## Results

Among the 113 children with BEEC referred to the large tertiary center, seven boys with PPE underwent the current modified surgical approach. The mean ± SD and median (range) of age at the time of the surgery were 6.5 ± 2.4 and 7 years (4–9 years), respectively. The family history of the patients was noncontributory. All the patients were incontinent (Grade 3) during the initial continence work-up. Regrading to the presence of primary VUR (grade 2 in all), three patients (Unilateral VUR in one and bilateral VUR in two) had a history of bulking agent injection prior to the reconstructive surgery. One patient who had a positive urine culture before the surgery was treated with antibiotics, confirmed with a second urine culture. The mean ± SD and median (range) of the follow-up period was 8.5 ± 6 and 7 years (2–23 years). None of the patients were lost during the follow-up.

**Fig. 2:**
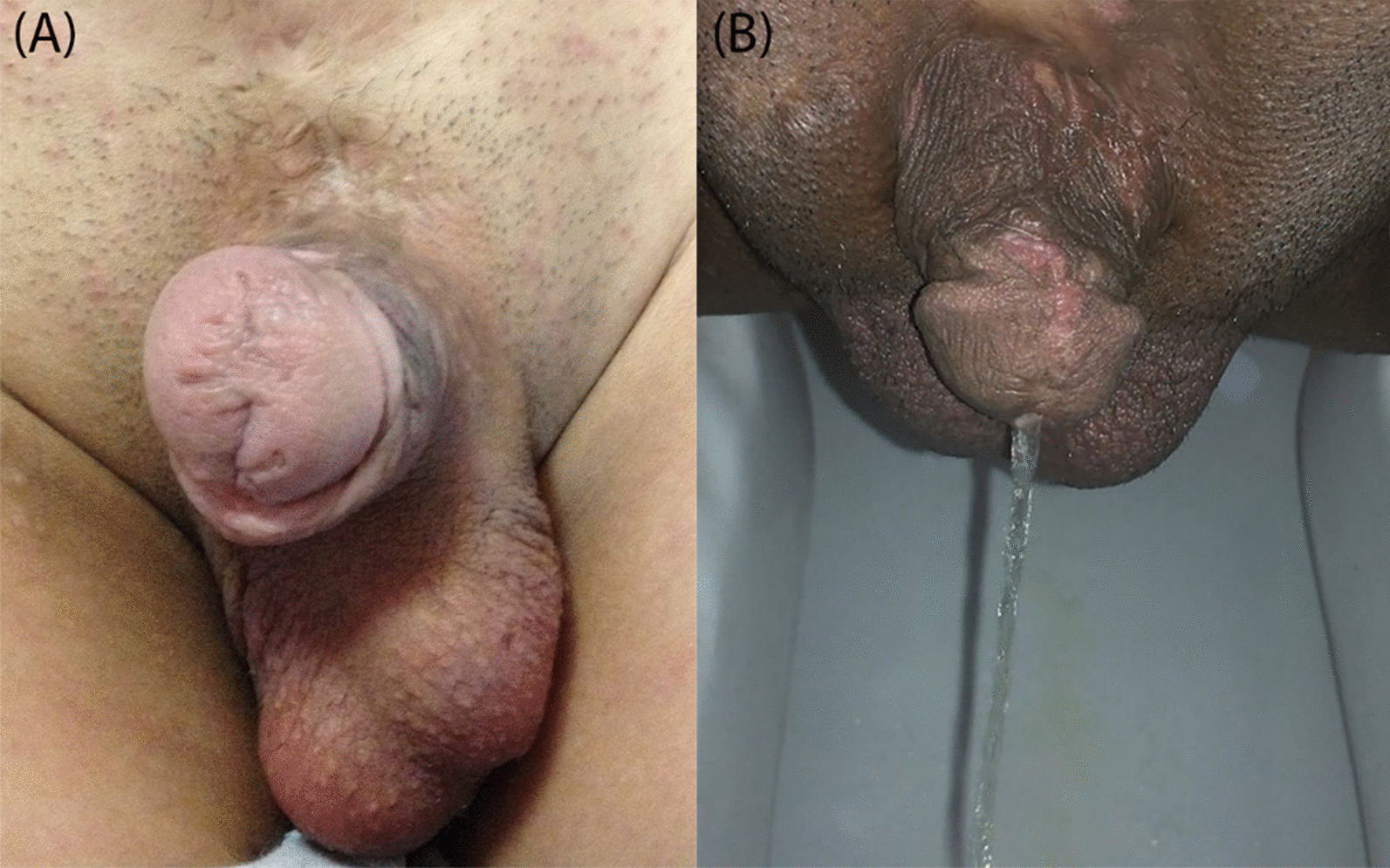
Long-term post-operation flaccid penis of **a** one mature patient, **b** another mature patient urinating

According to the intraoperative cystoscopic evaluations, all patients had incompetent striated sphincter, a wide opened BN, and normally configured ureteral orifices. The postoperative course was uneventful and major complications, including penile ischemia or hypospadias, did not occur. Moreover, common complications of urethral stricture, urethrocutaneous fistula, and dehiscence were not observed among the children in our small study group, except for a surgical site infection (Grade 2 the Clavien-Dindo classification) that was treated with antibiotics. Spontaneous catheter dislodgement did not occur in any patient. All children had normal volitional voiding per urethra, four achieved total dryness (Grade 0), and three had an infrequent urinary leakage following the strenuous activities (Grade 1). Ever since, according to the outcomes of the serial ultrasonography and VCUG, no complication was observed to be associated with the upper urinary tract. However, in the second year of follow-up in one boy, VCUG showed bilateral VUR (grade 2), resolved by the sub ureteric injection of the bulking agent within four months. The bladder capacity was mainly increased within the first year. According to the available urodynamic studies data, the mean ± SD of the bladder capacity augmented from 54.5 ± 11 to 124 ± 40.0 within 6 months (*P *< 0.05), and to 194 ± 47.5 at 18 months after surgery (*P *< 0.05). According to the examinations performed on the last follow-up and parent's survey, cosmetic outcomes were satisfactory (Fig. 2). None of our patients presented with chordee. Four of these children had normal erectile function and ejaculation according the obtained history on last follow-up regarding getting and maintaining an erection and subsequent ejaculation, while the others have not reached puberty yet. None of the patients reported involvement in a sexual relationship (Tables 1, 2).

**Table 1:** Individual Patients' characteristics before and after the modified single stage surgery of isolated penopubic epispadias in men

Patient nos.	Age at surgery (year)	F/U (years)	Continence Score^a^		Bladder capacity (ml)		
			Pre-op	Post-op	Pre-op	After 6 month	After 18 month
1.	9.1	7	3	0	53	140	275
2.	7.2	5.5	3	1	71	130	201
3.	2	2	3	1	37	64	140
4.	6	8.9	3	0	60	66	132
5.	4	5	3	1	52	146	167
6.	7	23	3	0	N/A	143	213
7.	9	8	3	0	N/A	180	229

N/A, not available; op, operation

^a^Based on a previously defined classification, which is also explained at methods

**Table 2:** Summation of characteristics and results

Variable	Number of patients (%)
*Patients' characteristics*	
Mean (SD) age at surgery	6.5 (2.4)
Mean (SD) years of follow-up	8.5 (6)
Children reached puberty	4 (57.1)
*Pre-operative complications*	
Dorsal curvature	7 (100)
Urinary incontinence (Grade 3)	7 (100)
Urinary tract infection	1 (14.3)
*Post-operative complications*	
Urethrocutaneous fistula	0 (0)
Urethral stricture	0 (0)
Surgical site infection^a^	1 (14.3)
Penile ischemia	0 (0)
Vesicoureteral reflux	1 (14.3)
Erectile dysfunction^b^	0 (0)
Abnormal ejaculation	0 (0)
*Post-operative outcomes*	
Continence	7 (100)
Grade 0	4 (57.1)
Grade 1^c^	3 (42.9)
Erection^b^	4/4 (100)
Ejaculation^b^	4/4 (100)

^a^Clavien-Dindo classification (grade 2)

^b^Among patients who reached puberty

^c^All patients are at prepubertal ages

## Discussion

The prevalence of psychosocial obstacles among epispadias patients is much higher compared to healthy people. An optimally effective surgery is a prerequisite for mitigating such problems. The inability of volitional voiding together with short dry intervals and dissatisfaction with genitalia and sexual function are the most annoying complaints that make the patients feel harassed, particularly in social interactions, and therefore should be addressed in surgical management of epispadias patients [3, 4, 6]. Although there are various methods established for the management of BEEC, most of our knowledge regarding the efficacy of these methods on patients with PPE are extrapolated from the postoperative outcomes of series including patients with BEEC, and there is a paucity of literature on the specific outcomes of surgical approaches performed on patients with PPE, as a particularly rare urogenital malformation [12, 13]. Herein, we report on the long-term results of a modified surgical technique performed by a single surgeon in a large referral center on seven boys with PPE. Patients underwent a single-stage combination of urethro-genitoplasty, bladder neck plication, and application of a pedicled fat pad flap to mitigate the complications of the contemporary adopted surgical techniques and to increase the continence rate among the patients with PPE. Nevertheless, since all our patients who experienced infrequent urinary leakage during strenuous activities were at prepubertal ages, there is the possibility of simultaneous improvement by pelvic muscles maturation and/or prostatic tissue growth following puberty [9].

Epispadias repair surgeries have evolved from Cantwell technique, later modified by Young, to Cantwell-Ransley and Mitchell-Bagli approaches over the course of more than a century. Each of these approaches is accompanied by its own complications [14–18]. Although the ability of volitional micturition was achieved by the contemporary surgical approaches with variable success rates, the results were still not impressive. Patients who achieved acceptable continence rates with current approaches had experienced severe postoperative complications or underwent more surgical interventions, which ultimately increased the mortality rate [8]. Patients who failed to attain continence after the initial surgery may require bulking agent injection and/or further surgeries, including one or more BNR(s) or augmentation cystoplasty. Although the injection of bulking agents is less invasive with reasonable outcomes, its effects are not permanent and the durability is shorter than the other techniques, necessitating repetitive injections [7, 8]. BNR is a commonly used surgical approach performed simultaneously with epispadias reconstruction or as a separate operation following the initial surgery. Gearhart et al. and Baka-Jakubiak performed a single-stage combination of epispadias repair and BNR with promising results with a continence rate of 69% and 89%, respectively. They were supportive of the notion that single-stage repair might be a more reasonable option than the conventional multi-stage reconstruction. They targeted sufficient bladder capacity as the prerequisite of a successful single-stage reconstruction [9, 10]. However, while the mean bladder capacity of our patients was almost half of the minimum amount reported in these two studies, our technique resulted in a significant increase in bladder volume with acceptable continence rates.

Patients with unsuccessful urine control after initial BNR may require other reconstruction surgeries and/or augmentation cystoplasty. Nonetheless, conducting additional surgeries on the BN not only decreases the possibility of achieving urine control, but also increases the rates of mortality. Capolicchio et al. and Peppas et al. advocated that fibrous scars developed following the multiple procedures performed at the BN decrease the possibility of ascertaining urinary continence in further surgeries [19, 20]. We do also believe that the most effective surgery in regards to attain continence for the management of isolated PPE is probably the first one. On the other hand, according to Braga et al., some patients with successful BNR have experienced irregular voiding patterns of irregular urine flow and increased post-voiding residuals, requiring clean intermittent catheterization [8]. Moreover, due to the inconsistency between the stimulation of the nervous system and the function of the reconstructed BN during ejaculation, abnormal ejaculation is one conceivable assumption in regards to the patients' infertility [21, 22]. Reddy et al. reported abnormal ejaculation in almost half of the patients who had undergone BNR [22]. Conduction of multiple BNRs may also increase the possibility of iatrogenic damages to seminal vesicles or vas deferens. Meanwhile, BNP using technique described primarily by Kelly in 1914 and applied previously by our surgeon in single-stage management of female epispadias [23, 24], produces acceptable bladder outlet resistance and preserves the innervations of the bladder. It bears mentioning that the bladder mucosa should not come into contact with suturing needle. Otherwise, the development of bladder stones may not be unlikely [25]. Additionally, owing to the better outcomes achieved with the use of non-absorbable sutures [24], we also performed BNP with the same stitches. In the present study, we performed urethra-bladder junction tubularization, first introduced by Ransley and Manzoni [26], prior to the bladder neck plication, and its competency was evaluated with manual hand compression after decatheterization. Another procedure we performed with the aim of increasing bladder outlet resistance was establishing a sling-like wide-based pedicled fat pad flap to the contralateral ventral aspect of the corpus spongiosum to provide an integrated ring support around the three main components at the proximal end of the penis, mimicking the function of the external sphincter. The constellation of urethra-bladder junction tubularization, bladder neck plication, and application of a pedicled fat pad flap culminated in acceptable bladder outlet resistance that significantly augmented bladder capacity within 6 and 18 months of follow-up. Additionally, the mitigated incidence of common postoperative complications compared to previous studies is presumably due to the adequate blood supply of corpus spongiosum preserved by the application of wide-based pedicled flaps.

It has been shown that complete penile disassembly may be associated with catastrophic complications, including penile loss due to glandular ischemia and/or corporal necrosis in some cases. Moreover, an animal study showed a significantly decreased endothelial and smooth muscle content, increased apoptosis, and upregulation of the TGF-β expression in the corpora cavernosa, presumably as a result of devascularization that occurred during complete penile disassembly [27]. Mentioned issues are the main suspects in regards to corporal loss and erectile dysfunction occurred following surgery. The preservation of blood flow has become of great importance in reconstructive surgery of epispadias [3]. Any disruption of blood supply may lead to tissue hypoxia and activation of pro-inflammatory cytokines, increasing the possibility of penile loss, urethral plate shortening, and reduction of corporal elasticity [27]. Furthermore, Acimi et al. showed shortening and narrowing of the urethral plate in all members of their study group, and Hafez et al. observed this phenomenon in one-third of their patients following complete penile disassembly [28, 29]. In the present study, we did not completely separate the corpora cavernosa and left the distal 1 cm attached. Moreover, mesothelial sparing during penile degloving and applying a pedicled fat pad flap were performed to provide better blood supply to the neo-urethra and corporal bodies. Besides, frequent examination of the glans blood flow after correction of the chordee was performed to assess any possible compression effects on NVBs. Providing the ability of removing the tightly sutured stitches and replacing them with the looser ones might be the superiority of this technique over cavernocavernostomy that was performed in the modified Cantwell-Ransley technique. Furthermore, peritubular tissue swelling of the neourethra and compression effects of the catheter may increase the possibility of venous congestion. Venous congestion is among the various causes of postoperative penile ischemia [30]. We hypothesized that recatheterization of the neourethra with a smaller-sized catheter could effectively address this issue. The details mentioned above are suggestive of the importance we placed on maintaining blood flow and avoiding devascularization at all stages, evidenced by developing no penile ischemia, urethrocutaneous fistula, urethral stricture, erectile dysfunction, or hypospadias in our patients.

The current study highlights the possible merits of our modified single-stage approach in the management of PPE. The only complications occurred consisted of an early surgical site infection managed by oral antibiotics, and grade 2 of bilateral VUR resolved with subureteric injection of bulking agents within four months. Presumably, the precise attention we paid to blood supply preservation, avoiding devascularization, and preventing extreme traumatization in each step, resulted in the minimal incidence of major postoperative complications, normal erectile function, and good cosmetic outcomes. Moreover, achieving an acceptable continence rate and successful ejaculation can be addressed by avoiding bladder denervation by performing BNP instead of BNR and urethra-bladder tubularization. The limited number of cases in this series, the retrospective design, and the advanced ages of the patients are the limitations of the current study. Considering the fact that our center is a tertiary center, and due to the poor socioeconomic status, patients are referred to us at older ages than those reported in the literature.

## Conclusion

The introduced single-stage technique seems to be a safe and effective approach for management of male patients with isolated PPE. The high continence rate and acceptable cosmetic outcomes along with the minimal morbidity and short hospital stay length support the introduction of this technique as a reliable method for management of this entity. The promising results of this paper suggest the consideration and further evaluation of this technique.

Our pediatric congenital urological anomalies treatment and management insight is that: “the best operation is the first complete reconstruction in single stage and not multi procedures repair”

## Data Availability

The datasets used during the current study along with images of the surgical technique is available from the corresponding author on reasonable request.
